# Network analysis of spread of SARS-CoV-2 within dialysis clinics: A multi-center network analysis

**DOI:** 10.1371/journal.pone.0299855

**Published:** 2024-03-08

**Authors:** Sunpeng Duan, Yuedong Wang, Peter Kotanko, Hanjie Zhang

**Affiliations:** 1 Department of Statistics and Applied Probability, University of California, Santa Barbara, Santa Barbara, California, United States of America; 2 Department of Medicine, Icahn School of Medicine at Mount Sinai, New York, New York, United States of America; 3 Renal Research Institute, New York, New York, United States of America; Warren Alpert Medical School of Brown University: Brown University Warren Alpert Medical School, UNITED STATES

## Abstract

**Background:**

In-center hemodialysis entails repeated interactions between patients and clinic staff, potentially facilitating the spread of COVID-19. We examined if in-center hemodialysis is associated with the spread of SARS-CoV-2 between patients.

**Methods:**

Our retrospective analysis comprised all patients receiving hemodialysis in four New York City clinics between March 12^th^, 2020, and August 31^st^, 2022. Treatment-level clinic ID, dialysis shift, dialysis machine station, and date of COVID-19 diagnosis by RT-PCR were documented. To estimate the donor-to-potential recipient exposure (“donor” being the COVID-19 positive patient denoted as “COV-Pos”; “potential recipient” being other susceptible patients in the same shift), we obtained the spatial coordinates of each dialysis station, calculated the Euclidean distances between stations and weighted the exposure by proximity between them. For each donor, we estimated the donor-to-potential recipient exposure of all potential recipients dialyzed in the same shift and accumulated the exposure over time within the ‘COV-Pos infectious period’ as cumulative exposures. The ‘COV-Pos infectious period’ started 5 days before COVID-19 diagnosis date. We deployed network analysis to assess these interactions and summarized the donor-to-potential recipient exposure in 193 network diagrams. We fitted mixed effects logistic regression models to test whether more donor-to-potential recipient exposure conferred a higher risk of SARS-CoV-2 infection.

**Results:**

Out of 978 patients, 193 (19.7%) tested positive for COVID-19 and had contact with other patients during the COV-Pos infectious period. Network diagrams showed no evidence that more exposed patients would have had a higher chance of infection. This finding was corroborated by logistic mixed effect regression (donor-to-potential recipient exposure OR: 0.63; 95% CI 0.32 to 1.17, p = 0.163). Separate analyses according to vaccination led to materially identical results.

**Conclusions:**

Transmission of SARS-CoV-2 between in-center hemodialysis patients is unlikely. This finding supports the effectiveness of non-pharmaceutical interventions, such as universal masking and other procedures to control spread of COVID-19.

## Introduction

Patients with end stage kidney disease (ESKD) are immunocompromised and thus particularly susceptible to infections, including severe acute respiratory syndrome coronavirus 2 (SARS-CoV-2). Hemodialysis patients have been impacted severely by coronavirus disease 2019 (COVID-19) with high morbidity and mortality rates (for review see [1]). Most ESKD patients are treated by thrice weekly in-center hemodialysis. This treatment modality entails frequent close interactions with other patients and clinic staff. For example, patients treated in consecutive dialysis shifts on the same day may share the same dialysis chair that may serve as a vector for the transmission of SARS-CoV-2. While plausible, this transmission pathway has been shown to be unlikely [2]. However, while receiving hemodialysis, patients also spend several hours near concurrently dialyzed fellow patients, raising plausible concerns that this setting may facilitate the spread of coronavirus disease 2019 (COVID-19) between patients. Providers of dialysis clinics have addressed this risk in multiple ways, including mandatory universal masking. Despite best efforts, some residual risks may remain. It is reasonable to hypothesize that the odds of spreading SARS-CoV-2 between patients are inversely correlated with the physical distances between patients while being dialyzed concurrently. To examine this hypothesis, we evaluated the risk of SARS-CoV-2 infection among patients undergoing dialysis simultaneously across four clinics. Our analysis specifically considered the Euclidean distance between dialysis chairs as a variable influencing the observed risk.

## Materials and methods

### Participants

We used data from all in-center hemodialysis patients treated at four New York City dialysis clinics operated by the Renal Research Institute (New York, NY, United States) between March 12^th^, 2020, and August 31^st^, 2022.

### Data

Treatment-level date, clinic ID, dialysis shift, and dialysis machine station number were documented in electronic medical records. There were three or four daily dialysis shifts. The station number indicates its physical location in the clinic where treatment occurs. The clinic’s digital floor plan allowed us to identify the spatial coordinates of each dialysis station. The dates of COVID-19 diagnosis by positive SARS-CoV-2 reverse transcription polymerase chain reaction (RT-PCR) tests were documented.

The study was conducted following a protocol reviewed by WCG Institutional Review Board (IRB) with the protocol number ES-21-004. WCG IRB determined that since the patient data was a limited data set, it was exempt from requiring informed consent.

Authors only had access to limited data. Authors had no access to information that could identify individual participants during or after data collection.

### Facility infection control procedures

The dialysis clinics’ standard mitigation strategies included screening patients for travel and COVID-19 exposure history, signs, and symptoms of infection prior to entry to the clinic, obtaining nasopharyngeal or oropharyngeal swabs, and performing testing for SARS-CoV-2 by RT-PCR in all patients with suspected COVID-19, designating isolation sections within facilities, and converting entire facilities to SARS-CoV-2 isolation units. Confirmed SARS-CoV-2 patients were required to dialyze in COVID-19 units or COVID-19 shifts. All patients were provided with and required to wear a surgical face mask throughout their time in the facility. Dialysis staff and physicians were required to wear surgical face masks, face shields, gowns, and gloves during all direct patient care activities. If treating known COVID-positive patients, dialysis staff, including physicians, N-95 masks were strongly advised if possible but not mandated because of intermittent shortage of N-95 masks.

### Statistical analyses

**Fig 1:**
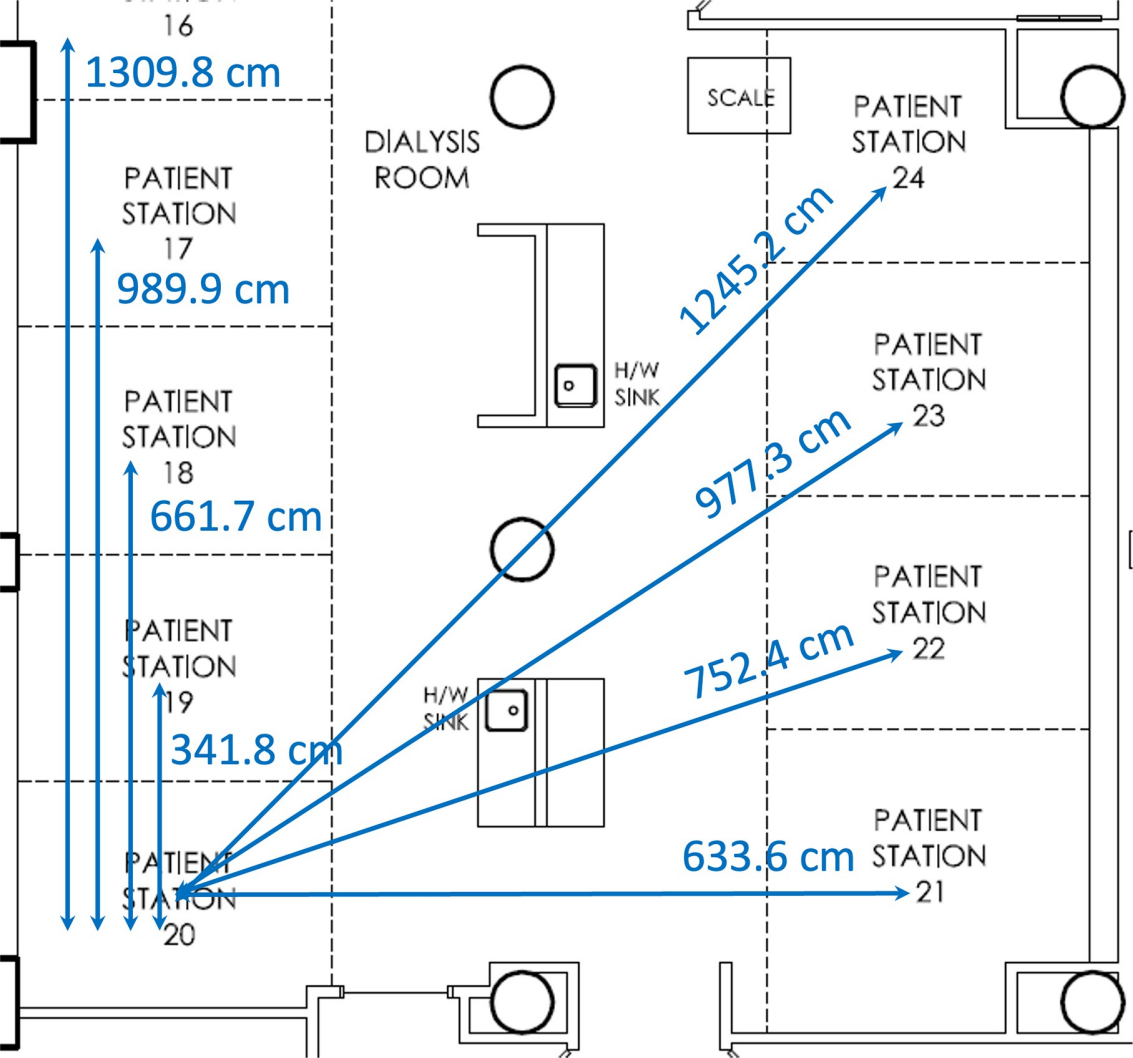
An illustration of Euclidean distances between stations. The Euclidean distances between station 20 and all other stations are shown. Only part of the clinic’s digital floor plan is shown here. All other paired distances were calculated and used for the analysis but are not displayed in the figure.

We performed a retrospective analysis to investigate whether the in-center SARS-CoV-2 transmission was associated with the donor-to-potential recipient (“donor” being the COVID-19 positive denoted as “COV-Pos” patient, “potential recipient” being other susceptible patients in the same shift) exposure. COV-Pos defines a patient with a positive RT-PCR test for SARS-CoV-2 of a nasopharyngeal or oropharyngeal swab. The ‘COV-Pos infectious period’ was defined as 5 days before COVID-19 diagnosis, as suggested by several reports [3, 4]. For each day during the ‘COV-Pos infectious period’, we first matched each “donor” patient with all “potential recipient” patients treated in the same dialysis shift at the same hemodialysis facility on the same date. We obtained each dialysis station’s actual location coordinates and calculated the Euclidean distances between them. An illustration of Euclidean distances between stations is shown in Fig 1.

**Fig 2:**
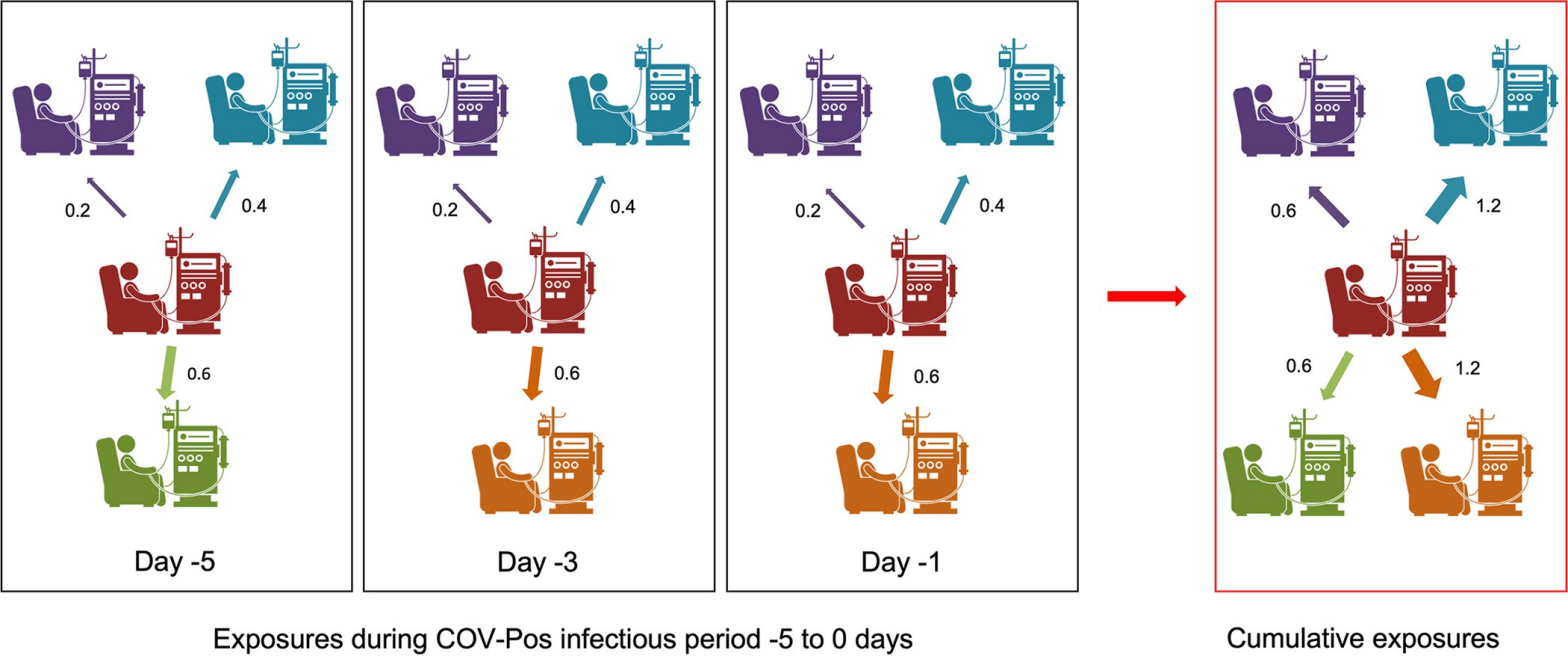
An illustration of exposure to one donor patient during the infectious period of potential recipients who received hemodialysis in the same shift. The red patient indicates the “donor” patient, the orange, blue, purple, and green patients are potential recipient patients dialyzed in the same shift on the same date, and the edges represent the donor-to-potential recipient exposure. The thickness of the line indicates exposure strength between donor and patient: the thicker the line, the higher the exposure value, and the closer the patient station is to the donor station. For example, on day -5, the green patient sits closest to the red donor patient, and the exposure value is 0.6, which is larger than 0.4 for the blue patient and 0.2 for the purple patient, two patients sit further from the red donor patient. Treatments on days -1, -3, and -5 relative to the “donor” patient’s COVID-19 diagnosis day are displayed for illustration. We accumulated exposure for each pair of patients by adding exposures during the infectious period. For example, the purple patient had three exposures, with 0.2 in each exposure and an accumulated exposure of 0.6; the orange patient had two exposures, with 0.6 in each exposure and an accumulated exposure of 1.2.

Then, the donor-to-potential recipient exposure was weighted by proximity between stations, i.e., the inverse Euclidean distance between dialysis stations. An illustration of exposure to one “donor” patient during an infectious period is shown in Fig 2, where the red patient indicates the “donor” patient, and the orange, blue, purple, and green patients are potential recipient patients dialyzed in the same shift on the same date, and the edges represent the donor-to-potential recipient exposure. The thickness of the line indicates exposure strength between donor and patient: the thicker the line, the higher the exposure value, and the closer the patient station is to the donor station. For example, on day -5, the green patient sits closest to the red donor patient, and the exposure value is 0.6, which is larger than 0.4 for the blue patient and 0.2 for the purple patient, two patients sit further from the red donor patient. Treatments on days -1, -3, and -5 relative to the “donor” patient’s COVID-19 diagnosis day are displayed for illustration. For simplicity, we only show three “potential recipients’’ stations around the “donor” patient station for each day. Note that “potential recipients’’ on different days could be different (e.g., the purple and the blue patients were dialyzed on their specific stations from day -5 to day -1, the green patient only appeared on day -5, the orange patient used that station on day -3 and day -1). The exposure between the same pair of patients increased due to several concurrent treatments. Therefore, we accumulated the exposure over time within the ‘COV-Pos infectious period’ as cumulative exposures, which is also described in Fig 2, the edges then represent the cumulative exposures. For example, the purple patient had three exposures, with 0.2 in each exposure and an accumulated exposure of 0.6; the orange patient had two exposures, with 0.6 in each exposure and an accumulated exposure of 1.2.

The follow-up period for each potential recipient patient was defined as 3 days after the first exposure and 10 days after the last exposure. A patient was identified as COV-Pos if tested positive for SARS-Cov-2 during the follow-up period; otherwise, the patient was identified as a COVID-19 negative (denoted as COV-Neg) patient. We also built mixed effect logistic regression models with the dataset generated from the network diagrams. In our models, the binary response was SARS-CoV-2 positivity/negativity of all potential recipient patients, while the explanatory variable was the exposure. The observations generated from the same network diagram were correlated due to the same SARS-CoV-2 ‘donor’ patient. Therefore, we added the random effect of the SARS-CoV-2 ‘donor’ patient ID. We fitted two models: Model 1 considers exposure as a continuous explanatory variable, and Model 2 categorizes exposure based on its quantiles (Exposure1: exposure ≤*Q*_1_; Exposure2: *Q*_1_<exposure ≤*Q*_2_; Exposure3: *Q*_2_<exposure ≤*Q*_3_; Exposure4: exposure >*Q*_3_), where *Q*_1_, *Q*_2_, and *Q*_3_ are the first, second, and third quantiles of the donor-to-potential recipient exposure. We also performed the same analyses before and after vaccination roll-out. Furthermore, we performed sensitivity analyses with a different follow-up period, starting 1 day after the first exposure and ending 14 days after the last exposure day.

## Results

We used data from all 978 in-center hemodialysis patients treated at four dialysis clinics. The mean and median number of treatments per patient per week are 2.597 and 2.886, respectively. During the study period, 287 (29.3%) out of these 978 patients tested positive for SARS-CoV-2. There were 94 (9.6%) COV-Pos patients who did not have contact with other patients during the COV-Pos infectious period, resulting in a cohort of 193 (19.7%) COV-Pos patients for further analysis. We counted the number of positive patients on each shift with at least one positive PCR test. In total 151 shifts with at least 1 positive PCR test. Among them, 122 shifts with only 1 positive PCR test, 19 shifts with 2 positive PCR tests, 8 shifts with 3 positive PCR tests, 1 shift with 4 positive PCR tests, and 1 shift with 5 positive PCR tests.

**Fig 3:**
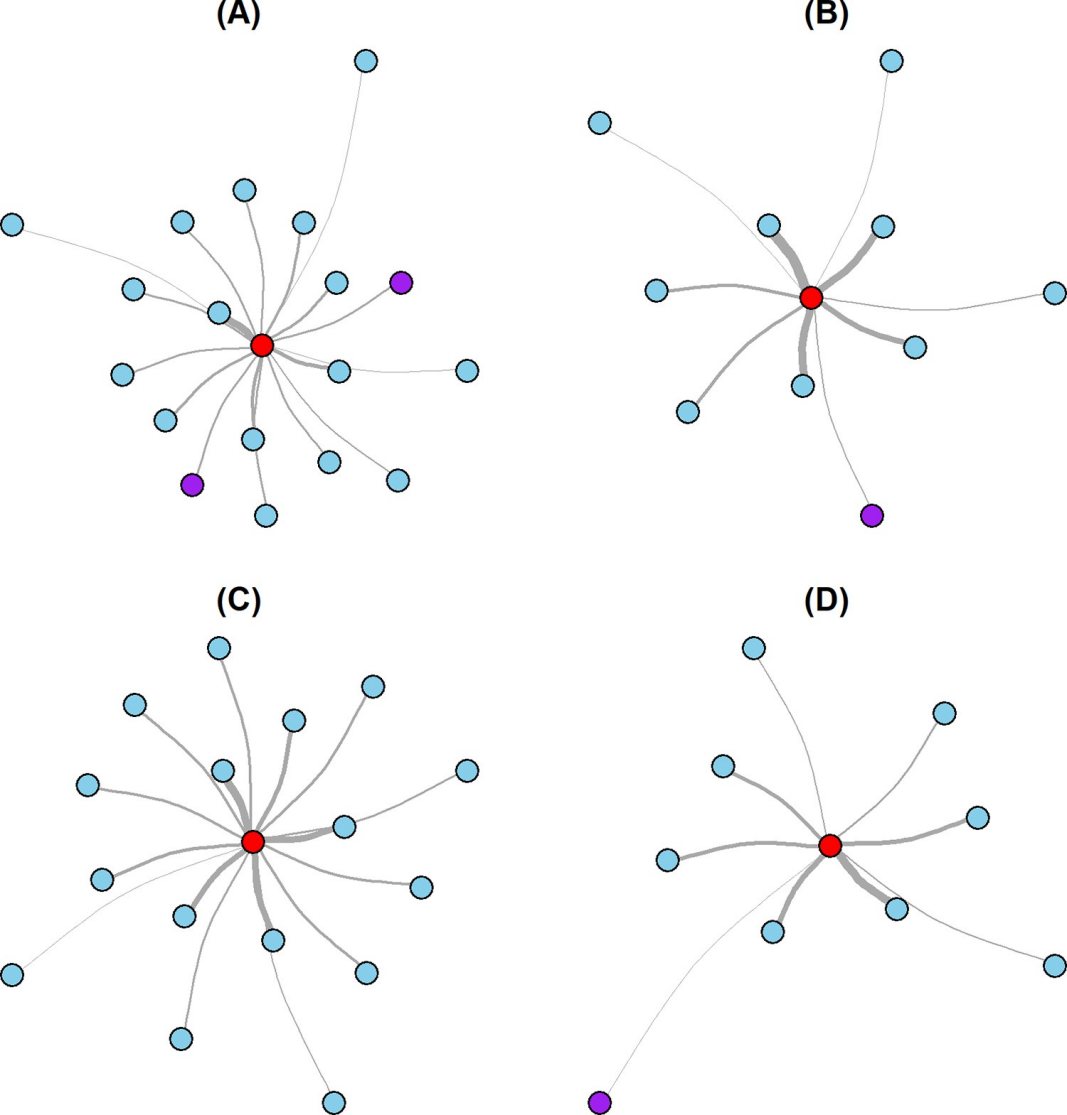
Network diagrams from four randomly selected SARS-CoV-2 ‘donor’ patients. The red central vertex represents the SARS-CoV-2 ‘donor’ patient, while other vertices represent COV-Neg ‘potential recipient’ patients (blue dots, tested negative for SARS-Cov-2 during the follow-up period) and COV-Pos ‘potential recipient’ patients (purple dots, tested positive for SARS-Cov-2 during the follow-up period). The edges represent the weighted donor-to-potential recipient exposure. The thicker the line width indicates more exposure to the donor patient. Two of these networks ((A) and (B)) are before the first vaccination date (2020-12-18), while the other two of these networks ((C) and (D)) are after the first vaccination date.

We summarized the donor-to-potential recipient exposure in 193 network diagrams with the defined ‘COV-Pos infectious period’. In each network diagram (see example diagram Fig 3), the red central vertex represents the ‘donor’ patient, while other vertices represent COV-Neg ‘potential recipient’ patients (blue dots, tested negative for SARS-Cov-2 during the follow-up period) and COV-Pos ‘potential recipient’ patients (purple dots, tested positive for SARS-Cov-2 during the follow-up period) who could have contracted SARS-CoV-2 from the ‘donor’ patient. The edges represent the weighted donor-to-potential recipient exposure. The line thickness indicates the exposure strength between the donor and the ‘potential recipient’ patient.

**Fig 4:**
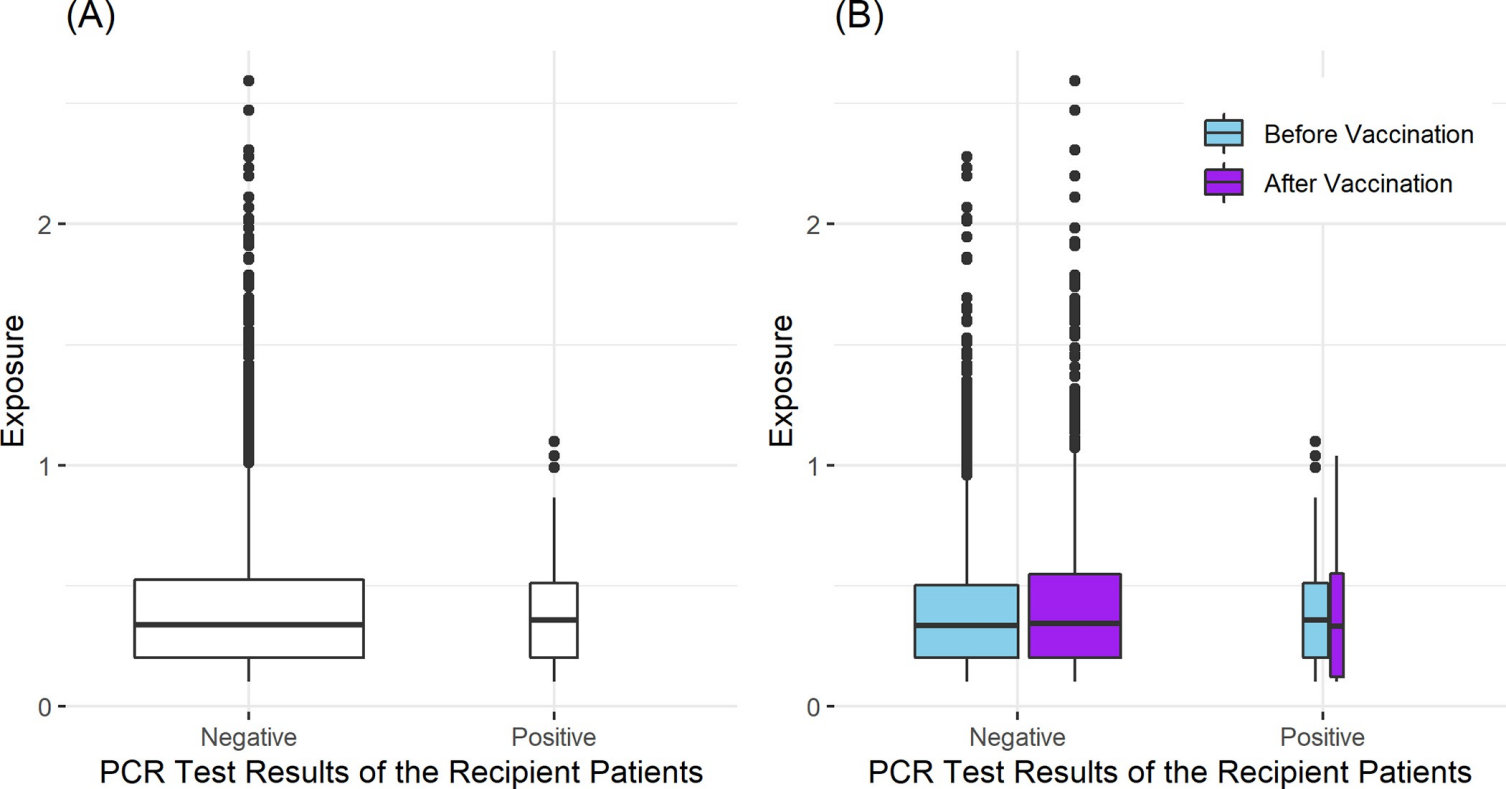
Boxplots of the donor-to-potential recipient exposure for the potential recipient patients tested SARS-CoV-2 positive or negative during the follow-up period. Panel (A) is the boxplot based on the overall dataset, while the boxplot in panel (B) separately summarizes the exposure before and after the vaccination.

We calculated the donor-to-potential recipient exposure for each SARS-CoV-2 ‘donor’ patient. The boxplot of the donor-to-potential recipient exposure for the recipient patients who tested SARS-CoV-2 positive or negative during the follow-up period is shown in Fig 4.

The first (*Q*_*1*_), second (*Q*_*2*_), and third (*Q*_*3*_) quantiles of the donor-to-potential recipient exposure were 0.2000, 0.3380, and 0.5227, respectively. We built two mixed effect logistic regression models and summarized the results in Table 1. Our data were generated according to each positive patient. A negative patient who is exposed to more than one positive patient in the same shift has multiple entries of exposure. Therefore, multiple exposures were accounted for when fitting linear mixed effects models.

**Table 1:** Mixed effect logistic regression results.

	Model 1			Model 2		
	Estimate	p-value	OR (95% CI)	Estimate	p-value	OR (95% CI)
Constant	-3.4233	< 10–4		-3.4518	< 10–4	
Exposure	-0.4659	0.163	0.63 (0.32, 1.17)			
Exposure1^a^ (Reference)				0		1
Exposure2^a^				-0.3738	0.137	0.69 (0.42, 1.12)
Exposure3^a^				-0.0411	0.860	0.96 (0.61, 1.52)
Exposure4^a^				-0.2423	0.329	0.78 (0.48, 1.27)
Variance of Random Effect	1.01			0.9886		

^a^ Exposure1: exposure≤*Q*_*1*_; Exposure2: *Q*_*1*_<exposure≤*Q*_*2*_; Exposure3: *Q*_*2*_<exposure≤ *Q*_*3*_; Exposure4: exposure> *Q*_*3*_, where *Q*_*1*_, *Q*_*2*_, and *Q*_*3*_ are the first, second and third quartile of the donor-to-potential recipient exposure.

In Model 1, the exposure was not significant (OR = 0.63, 95% CI 0.32, 1.17, *p* = 0.163), which indicated that more exposure did not increase the risk of in-center COVID-19 transmission. The results based on Model 2 were consistent with the results from Model 1. Exposure2 (OR = 0.69, 95% CI 0.42, 1.12, *p* = 0.137) and Exposure3 (OR = 0.96, 95% CI 0.61, 1.52, *p* = 0.860) and Exposure4 (OR = 0.78, 95% CI 0.48, 1.27, *p* = 0.329) were also not significant.

For these four clinics, the date when vaccination became universally available was 2020-12-18. Based on the donor’s positive date before or after 2020-12-18, we ran 2 analyses, one before and one after vaccination roll-out. We summarized our results in Table 2. Both before and after vaccination roll-out analyses lead to the same conclusion that transmission of SARS-CoV-2 between in-center hemodialysis patients is unlikely.

**Table 2:** Mixed effect logistic regression results before and after vaccination.

	Model 1			Model 2		
	Estimate	p-value	OR (95% CI)	Estimate	p-value	OR (95% CI)
	Before Vaccination					
Constant	-2.9695	< 10–4		-3.0401	< 10–4	
Exposure	-0.4198	0.280	0.66 (0.29, 1.35)			
Exposure1^a^ (Reference)				0		1
Exposure2^a^				-0.2350	0.397	0.79 (0.46, 1.36)
Exposure3^a^				-0.0678	0.801	0.93 (0.55, 1.58)
Exposure4^a^				-0.0801	0.769	0.92 (0.54, 1.58)
Variance of Random Effect	0.7807			0.7671		
	After Vaccination					
Constant	-4.0470	< 10–4		-3.8150	< 10–4	
Exposure	-0.3887	0.534	0.68 (0.17, 2.02)			
Exposure1^a^ (Reference)				0		1
Exposure2^a^				-0.9913	0.096	0.37 (0.10, 1.12)
Exposure3^a^				-0.5313	0.282	0.59 (0.21, 1.53)
Exposure4^a^				-0.1329	0.770	0.88 (0.35, 2.18)
Variance of Random Effect	0.4962			0.3774		

^a^ Exposure1: exposure≤*Q*_*1*_; Exposure2: *Q*_*1*_<exposure≤*Q*_*2*_; Exposure3: *Q*_*2*_<exposure≤ *Q*_*3*_; Exposure4: exposure> *Q*_*3*_, where *Q*_*1*_, *Q*_*2*_, and *Q*_*3*_ are the first, second and third quartile of the donor-to-potential recipient exposure.

We also conducted a sensitivity analysis with the follow-up period defined as 1 day after the first exposure and 14 days after the last exposure day and built two similar models. In Model 1, the exposure was significantly negative (OR = 0.39, 95% CI 0.21, 0.90, *p* = 0.0023). In Model 2, Exposure2 (OR = 0.99, 95% CI 0.68, 1.45, *p* = 0.969) and Exposure3 (OR = 0.76, 95% CI 0.51, 1.14, *p* = 0.187) were insignificant, while Exposure4 (OR = 0.62, 95% CI 0.40, 0.94, *p* = 0.027) was significantly negative. Both models indicate that more exposure did not increase the risk of in-center COVID-19 transmission.

## Discussion

In this research, we explored the transmission of COVID-19 among concurrently (i.e., in the same dialysis shift) dialyzed in-center hemodialysis patients. We first used network diagrams and boxplots as exploratory data analysis, then performed formal analysis using the mixed effects logistic regression model. Our findings indicate that patient-to-patient spread of COVID-19 is unlikely. Separate analyses by vaccination led to the same conclusions.

The study is relevant because patients with end stage kidney disease treated by dialysis are a highly vulnerable group that is severely impacted by COVID-19 [1]. During the COVID-19 pandemic, hemodialysis patients with COVID-19 infection were frequently hospitalized, and about 1 in 5 died from the infection [5]. There were reasonable concerns that COVID-19 may spread between patients, given their short physical distance while being dialyzed concurrently.

Our study complements research that explored the transmission of SARS-CoV-2 between two patients who dialyzed in consecutive shifts and sitting in the same dialysis chair [2]. This study found no evidence for such a transmission between patients. Taken together, the two studies do not support the hypothesis that patient-to-patient transmission of SARS-CoV-2 plays a significant role in the spread of COVID-19 among in-center hemodialysis patients. This conclusion is supported by a different line of research that investigated the presence of SARS-CoV-2 antibodies in the general population in New York City neighborhoods and hemodialysis patients who dwelled in those areas [6]. This study showed a strong association between seropositivity rates in the general population and dialysis patients, supporting the notion that COVID-19 infection in in-center maintenance HD patients can primarily be attributed to community-acquired infection.

Our study has some limitations. First, the Euclidean distance between dialysis stations does not necessarily represent the actual “functional” distance. For example, two dialysis stations with a short Euclidian distance between them may be in adjacent rooms, making a transmission less likely. While our analysis did not formally consider the layout of the clinic floor, based on our first-hand knowledge of the participating clinics, we can confidently confirm that the Euclidean distance between chairs is a very good representation of the “functional” distance. Second, in addition to being dialyzed in close proximity to each other, additional opportunities for contact between patients with and without SARS-CoV-2 infection exist, e.g., while patients spend time together in the clinic’s waiting area and/or during transportation in shared vehicles. These factors are not documented and, therefore, are almost impossible to model. Third, we cannot exclude that patients with asymptomatic and thus undiagnosed COVID-19 were recorded as non-COVID-19 patients. In that context, it is important to note that the viral load in asymptomatic COVID-19 patients is low compared to symptomatic patients, making them a source of infection less likely [5]. Fourth, we considered the use of an SIR model to test our hypothesis. However, the sample size is too small to provide trustworthy parameter estimates. Lastly, our analysis comprised four clinics in New York City that are run by one dialysis provider with consistently applied screening, testing, and facility infection control procedures. Therefore, the generalizability of our findings to other clinics and providers may be limited.

In summary, our analysis indicates that patient-to-patient transmission of SARS-CoV-2 between concurrently dialyzed in-center hemodialysis patients is unlikely. This finding supports the effectiveness of non-pharmaceutical interventions, such as universal masking and other procedures, to control the spread of COVID-19 among hemodialysis patients.
